# Anti-NMDA-receptor encephalitis: an unusual treatable cause of chronic insomnia?

**DOI:** 10.5935/1984-0063.20200080

**Published:** 2021

**Authors:** Wietse A. Wiels, Yacine Boudiba, Laura Seynaeve, Sylvie De Raedt

**Affiliations:** Vrije Universiteit Brussel, Centre For Neuroscience (C4N), Universitair Ziekenhuis Brussel, Department of Neurology - Brussels - Brussels - Belgium

**Keywords:** Anti-NMDA-Receptor Encephalitis, Insomnia, Immune-Mediated Encephalopathy, Limbic Encephalitis

## Abstract

We describe an unusual case of severe and chronic insomnia that proved to be eminently treatable. Initially presumed to be of primary psychiatric/toxicological origin, certain clinical and paraclinical clues led us to the diagnosis of NMDA-receptor encephalitis, an immune-mediated disease of the brain. Our patient responded dramatically to immunotherapy, effectively regaining normal sleep habits and significantly improving his general and mental health after 25 years of insomnia and drug abuse. Immune-mediated encephalopathies should be considered in the differential diagnosis of severe sleep disorders that present with additional neurological signs and symptoms, even when chronic.

## CASE REPORT

A 50-year old man presented to our emergency department. He had a history of chronic alcohol and hypnotic abuse. This resulted in multiple hospitalisations and emergency room visits for related issues such as pancreatitis with portal vein thrombosis, fall-related fractures, and possibly epileptic events linked to withdrawal attempts. He had seen several mental health professionals for addiction and depression, being on sick leave and under treatment with multiple psychoactive drugs for many years. The patient and his wife were adamant that his mental and toxicological issues were due to a subacute onset of profound insomnia some 25 years earlier.

After he suddenly stopped drinking and using zolpidem (>10 tablets per day), he experienced several episodes of alterations of consciousness as well as sudden ‘freezes’ in thinking and speech. These were diagnosed as withdrawal seizures and treated with diazepam.

During examination, eccentric and chaotic thinking was observed, without focal neurological signs. He manifested prolonged episodes of general fidgeting and unusual facial grimacing and stuttering sounds, with normal or only slightly impaired consciousness. These episodes had been present to a lesser degree for several years according to the patient and his wife. Blood pressure and heart rate were variable, his respiratory rate intermittently disordered. After additional generalized tonic-clonic seizures in the following hours, neither additional doses of diazepam nor valproic acid or levetiracetam were effective in stopping these seizures. Phenobarbital, however, significantly reduced the occurrence of these different paroxysms (speech/thought arrests, faciobrachial fidgeting, and generalized seizures).

**Figure 1:**
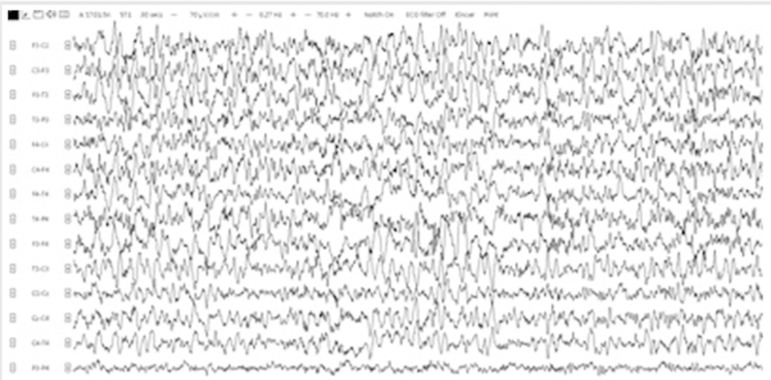
Electroencephalography shortly after presentation.

Laboratory analyses revealed an abnormal liver function, macrocytosis, hyperglycaemia, folic acid deficiency, and slight hyponatremia (129mmol/L). Electro-encephalography (EEG) demonstrated episodes of generalized slowing with bilateral frontotemporal rhythmic delta waves, often with superimposed beta (Figure 1). 48-hour EEG monitoring confirmed the near absence of physiological sleep (in combination with general slowing by diazepam use), without any clear seizure activity. Magnetic resonance imaging (MRI) of the brain did not show any abnormalities. Routine cerebrospinal fluid (CSF) analyses were normal.

Serum and cerebrospinal fluid (CSF) assays of N-methyl-d-aspartate-receptor (NMDA-R) antibodies, however, were highly positive. Positron-emission-tomography (PET) discovered no malignancy. We treated the patient with five consecutive daily doses of high-dose (1g) intravenous methylprednisolone and tapered him off all neuropsychiatric medications, with limited initial worsening of reported sleep quality during withdrawal of phenobarbital. This was alleviated by a slower taper. The patient refused further treatment with plasma exchange and did not show up for a polysomnographic evaluation in the acute phase.

At a follow-up visit three months later, the patient’s condition had markedly improved. He slept 7 hours every night, had stopped using alcohol and hypnotics, spontaneously lost 30 kilograms of weight without any major dietary changes, did not experience any psychological distress, and planned to return to work. Clinical neurological and mental evaluation were normal. Follow-up EEG’s were normal. Repeated serum tests did not show any remaining NMDA-R antibodies and no CSF controls were done.

## DISCUSSION

The combination of profound insomnia with epileptic events, brachiofacial choreiform fidgeting, autonomic instability, and general neuropsychiatric dysfunction in combination with diffuse EEG abnormalities made us consider the possibility of immune-mediated encephalitis in this patient, despite the remarkably long disease history.

These clinical findings echo the historical *agrypnia excitata* syndrome, which has only a few very specific causes, one of them being immune-mediated encephalopathies^1,2^.

Although alcohol and Z-drug withdrawal were also present - which often cause a similar picture of sleep disturbance with dysautonomia - a dramatic neuropsychiatric improvement with immunotherapy would not be expected in these conditions and does not explain the presence of antibodies in both serum and CSF.

Anti-NMDA-receptor encephalitis, arguably the most well-known and prevalent of the immune-mediated encephalopathies, is known to be associated to various disorders of sleep^3-6^. Often highly responsive to immunotherapy, it is a diagnosis that should not be missed in certain neuropsychiatric presentations. The illness may follow a relapsing-remitting course with neuropsychiatric sequelae in between episodes - possibly explaining the unusually long disease history in our patient^3-5^. We hypothesize that an initial bout of encephalitis triggered neuropsychiatric and sleep disorders, leading to abuse of alcohol and benzodiazepines that aggravated this situation, until a relapse of more florid ‘neurological’ symptoms led to a novel diagnosis as described above.

Several EEG abnormalities, often in the absence of electrographic seizures despite clinical attacks, have been described and hold prognostic significance^6,7^. In the appropriate clinical context, clinicians should consider the possibility of immune-mediated encephalitis in cases of severe insomnia, even when chronic.
